# Age‐related variations in prostaglandin E‐major urinary metabolite values in Japanese children

**DOI:** 10.1111/ped.70355

**Published:** 2026-03-04

**Authors:** Takatoshi Maeyama, Ayaka Takashiba, Ayaha Hata, Keinosuke Hizuka, Ryutaro Saura, Yuri Etani, Shin‐ichiro Hagiwara

**Affiliations:** ^1^ Department of Gastroenterology, Nutrition, and Endocrinology Osaka Women's and Children's Hospital Osaka Japan; ^2^ Fujirebio Inc. Tokyo Japan

**Keywords:** biomarker, child, reference values, ulcerative colitis

## Abstract

**Background:**

Prostaglandin E‐major urinary metabolite (PGE‐MUM) is an emerging noninvasive biomarker used to evaluate clinical and endoscopic activity in patients with inflammatory bowel disease. Previous studies have shown that PGE‐MUM values correlate with colonic inflammation in pediatric ulcerative colitis; however, reference values for children without inflammation have not been defined yet. This study aimed to determine the normal reference values of PGE‐MUM in healthy pediatric subjects without inflammatory conditions.

**Methods:**

Between December 2018 and January 2022, we prospectively enrolled 221 participants (cross‐sectional study, aged 0–15 years) who were undergoing growth hormone stimulation testing for diagnostic purposes and exhibited no symptoms of inflammation or elevated C‐reactive protein levels at a single pediatric center in Japan. PGE‐MUM values were measured using a chemiluminescent enzyme immunoassay and assessed for age‐ and sex‐related differences.

**Results:**

A total of 218 participants were included in the analysis. Based on age, the participants were classified as young children (2–6 years, *n* = 147) and older children (7–14 years, *n* = 66); the reference ranges for the two groups were identified as 18.4–58.7 μg/g·Cr and 12.4–50.3 μg/g·Cr, respectively. The PGE‐MUM values tended to decrease with increasing age; however, no significant sex‐related differences were observed (median 29.1 μg/g·Cr for boys versus 30.4 μg/g·Cr for girls).

**Conclusions:**

Healthy pediatric subjects generally have higher PGE‐MUM values than healthy adults, with particularly elevated values in young children (2–6 years). These observations suggest that age‐related variability must be taken into account when using PGE‐MUM as a biomarker in young children with ulcerative colitis.

## INTRODUCTION

According to the Selecting Therapeutic Targets in Inflammatory Bowel Disease (STRIDE)‐II initiative, the ultimate treatment goal for patients with inflammatory bowel disease (IBD) is deep healing, which includes clinical remission, complete endoscopic and histological recovery, and transmural healing.^1^ Adequate mucosal healing is known to reduce the risk of relapse,^1^ which is assessed using endoscopy; however, repeated endoscopic procedures are often challenging in children due to the burden of bowel preparation and the need for anesthesia.^2^ In such scenarios, biomarkers, including fecal calprotectin, which reflect mucosal activity, are extremely useful.^3^, ^4^ Prostaglandin E2 (PGE2), a key inflammatory mediator produced from arachidonic acid via cyclooxygenase (COX) enzymes, plays a central role in immune regulation, particularly in Th17‐type responses implicated in the pathogenesis of ulcerative colitis (UC).^5^, ^6^, ^7^ However, due to its rapid metabolism in the bloodstream, direct measurement of PGE2 is difficult.^8^ Prostaglandin E‐major urinary metabolite (PGE‐MUM; 7‐hydroxy‐5,11‐diketotetranorprosta‐1,16‐dioic acid) is a more stable form of PGE2 and can be measured noninvasively in urine samples.^9^, ^10^

As a promising biomarker, PGE‐MUM can be used to detect both endoscopic and histologic remission. A previous study demonstrated that urinary PGE‐MUM values strongly correlate with clinical activity (PUCAI: Pediatric Ulcerative Colitis Activity Index), endoscopic findings, and the extent of histologic inflammation.^11^, ^12^ Given its noninvasive nature, it is a practical alternative to endoscopy for continuous monitoring, especially in pediatric patients. However, despite its clinical relevance, reference ranges for PGE‐MUM have not yet been established for healthy children, limiting its broader application in pediatric care.

A previous study evaluated PGE‐MUM values in pediatric patients with functional gastrointestinal disorders (FGIDs) and the sample size was insufficient to establish reliable reference values.^11^ Therefore, the present study aimed to determine the standard range of urinary PGE‐MUM values in healthy children.

## METHODS

### Study design and subjects

In this single‐center cross‐sectional study, we prospectively enrolled participants under the age of 16 years who underwent growth hormone stimulation tests at the Osaka Women's and Children's Hospital, Japan, between December 2018 and January 2022. The exclusion criteria were: (1) presence of systemic inflammation or elevated C‐reactive protein (CRP) levels (the normal reference value for CRP is defined as less than 0.3 mg/dL), (2) presence of proteinuria, (3) history of diabetes, (4) current use of stimulant laxatives (e.g., sennosides) or nonsteroidal anti‐inflammatory drugs (NSAIDs), (5) history of colorectal surgery, and (6) pulmonary disorders.^10^, ^11^

After obtaining informed consent, their urine samples were collected by spontaneous voiding on the day of the stimulation test.

### Measurement of PGE‐MUM

The collected urine samples were promptly centrifuged at 1000 × *g* for 10 min, and the supernatant was stored at −30°C until analysis. PGE‐MUM concentrations were measured using a one‐step competitive immunoassay method based on chemiluminescent enzyme immunoassay technology and performed on the LUMIPULSE System (Fujirebio Inc., Tokyo, Japan).^11^ To account for the urine concentration variability, PGE‐MUM values were normalized to urinary creatinine and expressed as “μg/g·Cr.”

### Statistical analysis

All statistical analyses were performed using GraphPad Prism (version 10.4.0, GraphPad Software, San Diego, CA, USA). The Kolmogorov–Smirnov test was applied to assess the normality of the data distribution; if the *p*‐value was ≥ 0.05, the data were considered to be normally distributed. For normally distributed variables, the reference ranges were calculated as mean ± 1.96 standard deviation (SD), while the 95th percentile was used to define the reference range for non‐normal data distributions. The Mann–Whitney *U* test was used to compare the two study groups, and a *p*‐value of <0.05 was considered statistically significant.

### Age‐related analysis of urinary PGE‐MUM levels

To assess age‐associated variation in urinary PGE‐MUM levels, participants were categorized into two prespecified age strata (2–6 years and 7–14 years), informed by an apparent inflection in the data distribution observed on preliminary box plot analysis.

For children aged 2–6 years, linear regression analysis was performed to assess age‐dependent trends, and Kruskal–Wallis testing was additionally conducted to compare distributions among individual ages. In children aged 7–14 years, for whom no visually evident monotonic pattern was observed, age‐related differences were evaluated using the nonparametric Kruskal–Wallis test across subgroups. All p‐values were two‐sided, and a p‐value of <0.05 was considered statistically significant.

### Ethical considerations

This study was conducted in accordance with the ethical principles of the Declaration of Helsinki and complies with the Good Clinical Practice guidelines. The study protocol was reviewed and approved by the Ethics Committee of Osaka Women's and Children's Hospital (registration number: 1194). Prior to enrollment, written informed consent was obtained from the parents or legal guardians. When appropriate, assent was also obtained from the patients.

## RESULTS

### Subject characteristics

**FIGURE 1:**
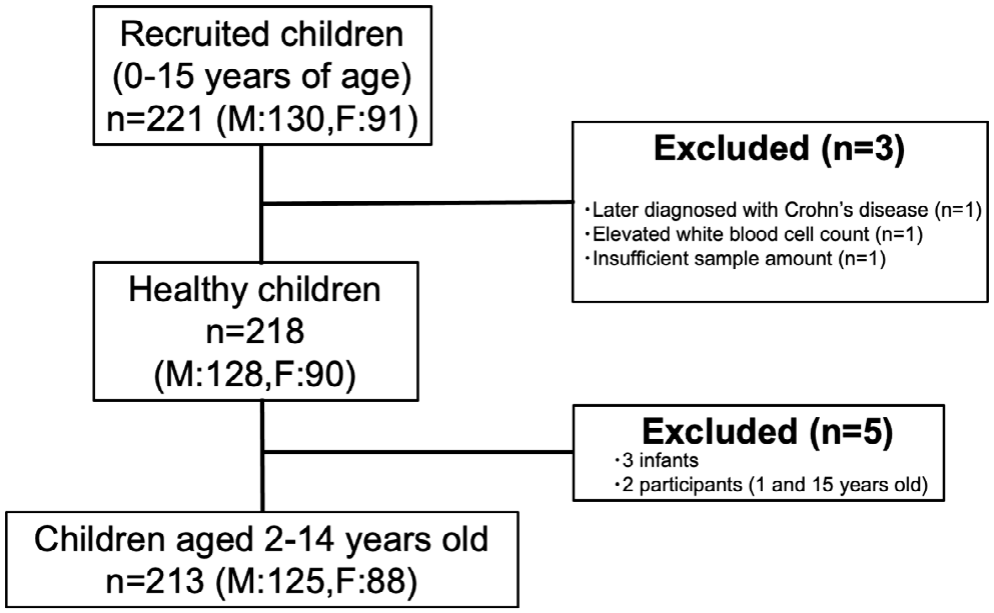
Flowchart of patient selection. Of 221 participants enrolled, 213 were included in the final analysis. The final cohort comprised 147 younger children (2–6 years; 85 boys, 62 girls) and 66 older children (7–14 years; 40 boys, 26 girls).

A total of 221 children (130 boys and 91 girls) were initially recruited. After applying the exclusion criteria, 218 children (128 boys and 90 girls) remained eligible. Following the exclusion of five participants (three infants and one each aged 1 and 15 years) from age groups with limited case numbers, 213 participants were included in the final analysis. In this cohort, 147 children (85 boys and 62 girls) were classified as younger children (2–6 years), and 66 (40 boys and 26 girls) as older children (7–14 years) (Figure 1). Participant characteristics are summarized in Table 1. The three infants in our study (an 8‐month‐old boy, a 10‐month‐old boy, and a 7‐month‐old girl) exhibited markedly elevated PGE‐MUM values (68.5 μg/g·Cr, 77.4 μg/g·Cr, and 92.5 μg/g·Cr, respectively) compared with those in the other age groups. Because infants represent a physiologically distinct age category and their extremely elevated PGE‐MUM values would skew the distributional characteristics of the older age groups, they were excluded from the primary statistical analyses. Detailed data for infants are provided in Table S1.

**TABLE 1:** Participant characteristics.

Age group	*N* (male/female)	Age (mean ± SD) (male/female)
Total	213 (125/88)	5.7 ± 3.1 (5.8 ± 3.3/5.4 ± 2.9)
2–6 years old	147 (85/62)	3.9 ± 1.3 (3.9 ± 1.3/3.9 ± 1.4)
7–14 years old	66 (40/26)	9.7 ± 2.2 (10.0 ± 2.1/9.2 ± 2.3)

### Age‐specific distribution of PGE‐MUM values

**FIGURE 2:**
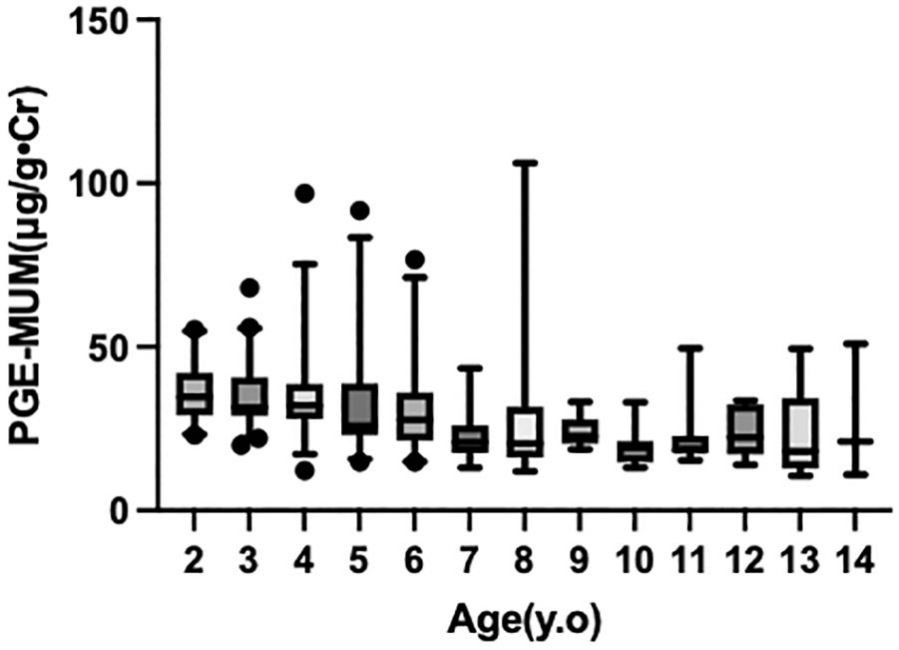
The age‐specific distribution of the PGE‐MUM values is shown using box‐and‐whisker plots. PGE‐MUM values declined with age in children under 7 years, with infants showing the highest levels. No clear age‐related trend was observed in older children (7–14 years).

Figure 2 illustrates the age‐specific distribution of the PGE‐MUM values using box‐and‐whisker plots. Among the younger children group (2–6 years), the PGE‐MUM values tended to decrease with increasing age; the median values also gradually declined, forming a smooth downward slope up to approximately under 7 years of age; however, no clear age‐related trend was observed in the older children group (7–14 years).

### Age‐related statistical analysis of PGE‐MUM values

To quantitatively assess age‐related trends, linear regression analysis was performed for children aged 2–6 years. Although the slope indicated a negative trend with increasing age, the association did not reach statistical significance (slope = −1.15, 95% CI −2.82 to 0.52; *p* = 0.18; *R* ^2^ = 0.01). A Kruskal–Wallis test comparing individual ages within this group also showed no statistically significant differences (*p* = 0.085), although a stepwise decline in median values was observed. In contrast, among children aged 7–14 years, the Kruskal–Wallis test demonstrated no significant differences across ages (*p* = 0.806), confirming the absence of age‐related variation in this older age group. Taken together, these findings indicate a biphasic pattern in urinary PGE‐MUM levels, characterized by a gradual decline during early childhood followed by a stable plateau from approximately 7 years onward.

### Sex‐related differences in PGE‐MUM values

**FIGURE 3:**
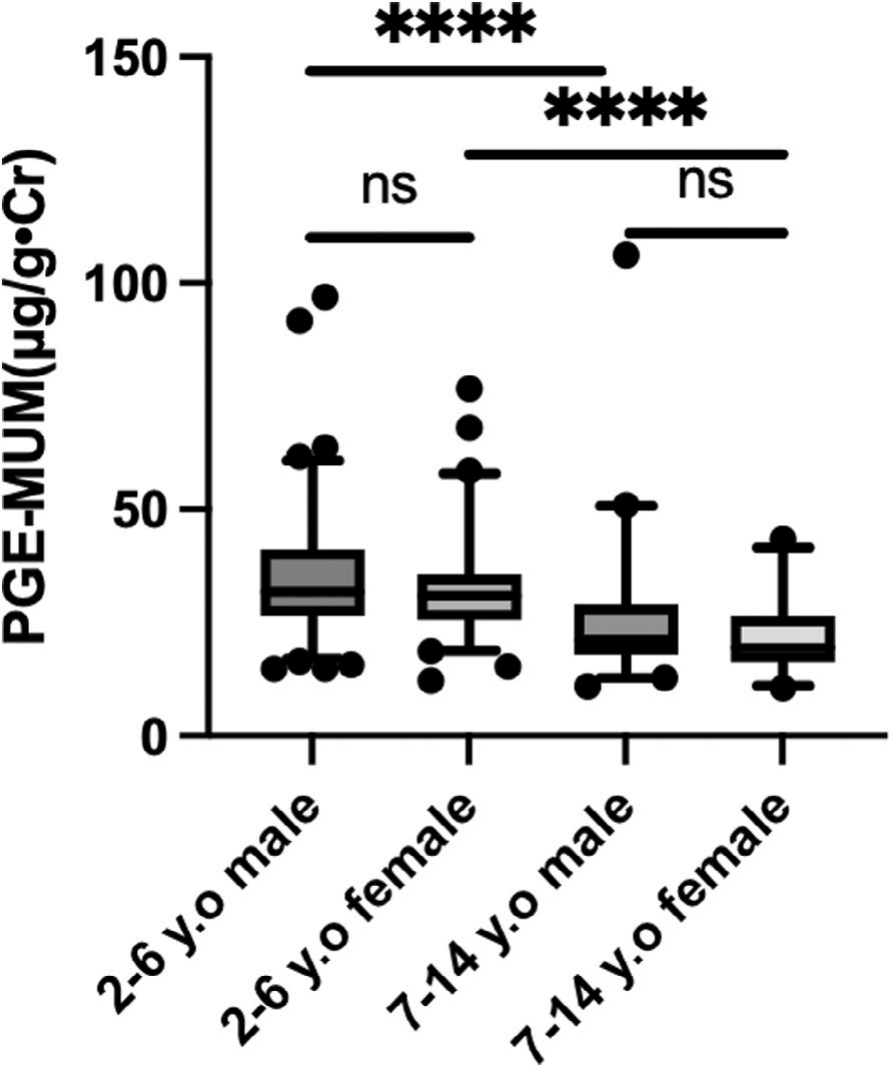
Age‐related comparison of PGE‐MUM values by sex. Sex‐related differences in PGE‐MUM values were not statistically significant across the cohort or within age groups. Median values were similar between boys and girls, but significantly higher in younger children than in older children for both sexes. ****p < 0.001.

Figure 3 demonstrates the sex‐related differences in the PGE‐MUM values in the study cohort. Across the entire age range, there was no statistically significant difference in the PGE‐MUM values between boys and girls (median 29.1 μg/g·Cr versus 29.9 μg/g·Cr; *p* = 0.45). Likewise, when divided into younger and older groups, no significant sex‐related differences were observed in the median PGE‐MUM values. In the younger group, the median value was 31.8 μg/g·Cr in boys and 30.9 μg/g·Cr in girls, whereas in the older group, the corresponding values were 21.2 μg/g·Cr and 19.4 μg/g·Cr, respectively. However, when comparing the two age groups, the PGE‐MUM values were significantly higher in the younger group than in the older group in both sexes (*p* < 0.001).

### Age‐wise reference range for PGE‐MUM in healthy children

The reference ranges were calculated separately for each age group. In the younger group, due to the non‐normal distribution, the 95th percentile values yielded a reference range of 18.4–58.7 μg/g·Cr. In the older group, due to the non‐normal distribution, the 95th percentile values yielded a reference range of 12.4–50.3 μg/g·Cr.

## DISCUSSION

PGE‐MUM is used as a biomarker for both the clinical and endoscopic activity of IBD in children and adults^11^, ^12^, ^13^; however, the normal values of PGE‐MUM have not been established in healthy children. In this study, we revealed the normal range of PGE‐MUM in healthy children without any symptoms or signs of inflammation. Given that PGE‐MUM offers a noninvasive, rapid, and simple method for monitoring inflammation, our findings provide important insights from a pediatric perspective.

Using data from 213 children, we observed that PGE‐MUM values were generally higher in children than previously reported in adults (95% confidence intervals: 8.7–42.3 μg/g·Cr).^14^ These results highlight the need for establishing pediatric‐specific reference ranges, as proposed in the present study for children without signs of inflammation.

The PGE‐MUM values observed in the study showed considerable inter‐subject variability. To elucidate potential contributing factors, we assessed three principal domains: age, sex, and other potential biological parameters. First, children exhibited higher PGE‐MUM values than adults,^14^ possibly due to age‐dependent differences in PGE2 metabolism. Circulating PGE2 levels are regulated by a balance between its production via COX‐1 and COX‐2 enzymes and its degradation by 15‐hydroxyprostaglandin dehydrogenase (15‐PGDH). The lungs serve as the primary site of PGE2 clearance through 15‐PGDH; and the kidneys also express this enzyme.^15^ In young children, these organs are functionally immature, potentially leading to inefficient PGE2 metabolism and elevated PGE‐MUM levels.

We also proposed the normal range based on the 97.5th percentile. The age‐dependent pattern of PGE‐MUM values observed in this study resembles that previously reported for fecal calprotectin, which also demonstrates age‐related decline.^16^, ^17^, ^18^, ^19^, ^20^

Another important finding was the steep decline in PGE‐MUM values in children under 6 years of age, followed by a more gradual decrease after 7 years. Although linear regression analysis in the 2–6 year group showed a negative age‐related trend, the association did not reach statistical significance, likely due to the considerable intra‐age variability and the limited resolution of integer‐based age categories. Likewise, when individual ages within the younger cohort were compared using the Kruskal–Wallis test, the result was only marginally significant (*p* = 0.085), suggesting a progressive rather than distinctly segmented decrease. In contrast, among children aged 7–14 years, the Kruskal–Wallis test demonstrated no significant differences across ages (*p* = 0.806), confirming that PGE‐MUM levels remain essentially stable during later childhood. Taken together, these results indicate that PGE‐MUM follows a biphasic age trajectory, characterized by a modest age‐related decline during early childhood and a subsequent plateau from approximately 7 years onward.

This biphasic pattern highlights the difficulty of establishing a single reference range for the entire pediatric population; accordingly, we divided the participants into younger (2–6 years) and older (7–14 years) age groups. These findings highlight the importance of considering the patient's age when interpreting PGE‐MUM values in pediatric patients.

Second, PGE‐MUM values tended to be higher in males compared to females. Previous studies have suggested that steroid sex hormones may influence PGE‐MUM in adults.^15^ While a similar trend was seen in our cohort, the difference was not statistically significant, suggesting that sex‐specific reference ranges may not be necessary in the clinical evaluations of children.

Third, genetic factors may also contribute to inter‐subject variability. It has been noted that individuals with homozygous mutations in the *15‐PGDH* gene, which encodes the primary prostaglandin‐degrading enzyme, show markedly reduced PGE‐MUM excretion.^21^ In addition, pathological variants of the *SLCO2A1* gene, which encodes a prostaglandin transporter, have been linked to elevated PGE‐MUM.^22^ Presumably, subclinical genetic polymorphisms may have similarly influenced inter‐subject variability observed in our cohort.

The three infants in our study had markedly elevated PGE‐MUM values. A previous report of 14 healthy infants showed a PGE‐MUM range of 47–155 μg/g·Cr, with a mean value of 82.1 μg/g·Cr,^23^ consistent with our findings. These results suggest that infants may require separate reference considerations due to their unique physiology. Accordingly, infants were excluded from the primary statistical analyses in order to avoid distorting the age‐related distribution pattern observed in older children; however, their individual data are presented in the Supplementary Table S1 for transparency.

In our previous study, we evaluated PGE‐MUM as a biomarker of endoscopic and histologic remission in 104 pediatric patients with UC (aged 6–16 years) and 39 controls with FGIDs.^11^ The median PGE‐MUM level in the control group (18.6 μg/g·Cr) closely matched the values observed for healthy children aged 7–14 years in the current study (21.2 μg/g·Cr in boys and 19.4 μg/g·Cr in girls). In contrast, the median and interquartile range (IQR) values for patients with UC were 28.5 and 16.2–46.3 μg/g·Cr, respectively. Although these values were higher than those for controls (median [IQR]: 18.6 [15.7–22.6] μg/),^11^ more than half of the UC patients still fell within the pediatric reference range (12.4–50.3 μg/g·Cr) for ages 7–14 years, suggesting that PGE‐MUM may not be appropriate as a diagnostic marker to differentiate patients with and without UC. Accordingly, we advocate the use of PGE‐MUM as a noninvasive biomarker for monitoring disease activity in pediatric patients diagnosed with UC.

This study has several limitations. First, all samples were collected from a single center, which may introduce sample bias. Second, we did not evaluate genetic variants, such as those in *15‐PGDH* or *SLCO2A1*, that could potentially affect PGE‐MUM metabolism.^22^ To enhance the generalizability of our findings, future multicenter studies with larger, more diverse populations and genetic profiling are warranted. Nevertheless, the strength of this study lies in establishing a foundational pediatric reference range for PGE‐MUM, supporting its future clinical application in monitoring disease activity in children with IBD.

In conclusion, this study demonstrated that PGE‐MUM values are generally higher in pediatric populations than in adults. While establishing age‐specific reference ranges for urinary PGE‐MUM values in healthy children, we observed that PGE‐MUM values showed a clear age‐related decline, particularly around the age of 6. While certain sex‐related differences were observed, these differences were not statistically significant. Based on these findings, we propose reference ranges stratified by age for younger (2–6 years) and older (7–14 years) children. Given its noninvasive nature and association with inflammatory activity, PGE‐MUM serves as a promising biomarker for monitoring disease activity in pediatric patients with IBD. Further multicenter studies are warranted to validate the proposed reference ranges and better understand the factors contributing to inter‐subject variability in the PGE‐MUM values.

## AUTHOR CONTRIBUTIONS

S‐i.H and T.M. designed the study; T.M., A.H., K.H., and R.S. assisted with data collection. S‐i.H and T.M. analyzed data; A.T. provided resources and supervised laboratory procedures; S‐i.H and T.M. wrote the manuscript; Y.E. gave conceptual advice. All authors read and approved the final manuscript.

## FUNDING INFORMATION

This work was partially funded by Fujirebio Inc.

## CONFLICT OF INTEREST STATEMENT

Ayaka Takashiba is an employee of Fujirebio Inc. The company had no role in the study design, data collection, analysis, or interpretation, manuscript writing, or the decision to submit the paper for publication.

## Supporting information


Table S1.

## Data Availability

The data that support the findings of this study are available from the corresponding author upon reasonable request.
