# Supplementary material for: Age‐related variations in prostaglandin E‐major urinary metabolite values in Japanese children
Source: Pediatr Int. 2026 Mar 4;68(1):e70355. doi: 10.1111/ped.70355 (PMC12961369; doi:10.1111/ped.70355)
Supplement: Supplementary file 1 — Table S1. [file PED-68-e70355-s001.docx]

**Table S1 PGE-MUM concentrations in infants**

| Case No | Age (months) | Sex | PGE-MUM(μg/g・Cr) |
| --- | --- | --- | --- |
| 1 | 7 | Female | 92.5 |
| 2 | 8 | Male | 66.0 |
| 3 | 10 | Female | 87.4 |
